# The natural course of hereditary angioedema in a Chinese cohort

**DOI:** 10.1186/s13023-020-01526-1

**Published:** 2020-09-22

**Authors:** Yang Cao, Shuang Liu, Yuxiang Zhi

**Affiliations:** 1 Department of Allergy & Clinical Immunology, Peking Union Medical College Hospital, Peking Union Medical College & Chinese Academy of Medical Sciences, National Clinical Research Center for Immunologic Diseases, #1 Shuaifuyuan, Wangfujing, Beijing, 100730 P.R. China; 2 School of Clinical Medicine, Chinese Academy of Medical Sciences & Peking Union Medical College, Beijing, 100005 China

**Keywords:** Hereditary angioedema (HAE), Natural course, Risk factors

## Abstract

**Background:**

Hereditary angioedema (HAE) is a rare disease with potential life-threatening risks. To study the natural course of HAE under therapy-free conditions throughout patient life is essential for practitioners and patients to avoid possible risk factors and guide treatment.

**Objectives:**

Describe the natural course of HAE and explore possible risk factors, providing new clues for guiding clinical prevention and treatment.

**Methods:**

A web-based survey was conducted in 103 Chinese patients with type 1 HAE. Disease progression at different age stages was provided by each participant. The data for exploring the natural course of HAE composed of two parts: one came from the participants who had never adopted any prophylactic drug for HAE; the other was from the patients with a history of medication, but only the periods before they got confirmed diagnosis and received medications were analyzed. The demographic characteristics, lifestyles, disease severity, and family history were also collected.

**Results:**

Among 103 patients, 14 (13.6%) had their first HAE attack before 10 years old and 51 (49.5%) between 10 and 19. The disease worsened in 83.3% of the patients in their twenties. The proportion of patients with symptoms alleviated increased after the age of 30 years old, but the disease maintained relatively severe in most cases before 50. The participants also reported 233 members shared similar symptoms of angioedema in their family and 30 had died of laryngeal edema with the median death age of 46 years old. The disease severity was not observed to be affected significantly by gender, BMI, alcohol or smoking.

**Conclusions:**

We summarized HAE progression patterns under therapy-free conditions, showing the natural course of HAE development along with aging. Long-term prophylaxis and symptomatic treatment are recommended for all HAE patients, especially young and middle-aged and might be adjusted depending on the disease progression.

## Background

Hereditary angioedema is a rare, autosomal dominant disease and is characterized by unpredictable and recurring episodes of subcutaneous and submucosal edema, which may affect the face, extremities, trunk, genitals, upper airways and gastrointestinal tract [1]. Most attacks are self-limiting, but abdominal edema may cause severe pain, nausea, diarrhea, and vomiting. Patients may even die of asphyxiation due to laryngeal edema in the absence of timely treatment [2].

A series of gene loci have been found to be associated with the pathogenesis of HAE [3–5]. Hereditary angioedema with C1 inhibitor deficiency (C1-INH-HAE) is caused by mutations in the *SERPING1* gene (OMIM 106100), the C1-INH encoding gene. C1-INH-HAE can be subdivided into type 1 and type 2 HAE. Type 1 HAE with low C1-INH expression and function accounts for the majority of HAE patients in China. A minority of Chinese patients developed type 2 HAE with normal levels but dysfunctional C1-INH protein [6]. Decreased plasma level of functional C1 inhibitor is insufficient to regulate the contact system, causing uncontrolled generation of bradykinin, a vasoactive peptide that increases vascular permeability by binding to and activating the bradykinin B2 receptor. Other types of HAE due to the defects in factor XII (HAE-FXII), plasminogen (HAE-PLG) or angiopoietin-1 (HAE-ANGPT1) have not been reported in China to date.

The patient populations are heterogeneous as regards to the frequency, severity, and locations of edematous attacks, presenting large intra- and inter-individual variation and are easily confused with other more common forms of recurrent angioedema subtypes. Based on the variety of clinical manifestations and rare clinical cases, previous data have shown a significant diagnostic delay of 8–10 years for type 1 HAE in Europe and the United States [7–9]. And the mean delay in diagnosis of HAE for Chinese patients can be as long as 12.64 years according to a 2013 survey [10]. Long diagnostic delay and inappropriate treatment will aggravate the humanistic and economic burden for patients [2, 11–13]. Danazol (an attenuated androgen) is the main drug for long-term prophylaxis in mainland China. There are also some patients who have received a confirmed diagnosis of HAE but refuse to take danazol, due to the side effects of the drug and relatively mild symptoms. And it is inconvenient to buy danazol in some regions. Patients with long diagnostic delay or adopting no preventive drugs are surveyed in our study to analyze the natural course of HAE.

The aim of this study was to describe the natural course of HAE throughout patient life and explore the possible risk factors under therapy-free conditions, which may provide some new insight into the clinical practice and its pathogenesis.

## Methods

### Subject

One hundred and three Chinese patients from 94 unrelated families who presented to the Department of Allergy and Clinical Immunology, Peking Union Medical College Hospital from 1983 to 2017 with a confirmed diagnosis of HAE type 1 were contacted through medical records. The diagnosis of HAE was based on the medical history and laboratory tests: (1) a history of recurrent skin angioedema without urticaria, and/or recurrent attacks of abdominal pain and vomiting, and/or laryngeal edema; (2) Repeated confirmation of lower levels of C4, C1-INH antigen and C1-INH function in serum. A web-based questionnaire was sent to all participants. If the data feedback was incomplete or ambiguous, the participant was contacted for specific clarification. The study protocol was approved by the research and ethics board of the Peking Union Medical College Hospital. The participants were informed of the objectives, the agency conducting the research and privacy protection of this survey. Informed consent was obtained from all patients before the questionnaires were completed.

### Questionnaire

The questionnaire was developed by using the wjx online survey platform (Changsha Ranxing Information Technology Co., Ltd., Changsha, P.R.C.). Demographic characteristics, disease progression description, and possible risk factors were contained in the survey. Close-ended questions with defined response categories were adopted in the part of the disease progression description. The participants were asked to choose one of the descriptions for the disease status at an interval of 10 years since birth. The descriptions included “silent stage”, “first episode”, “exacerbation stage”, “stable stage” and “remission stage”. Frequency, location, average edema resolution time and records of lifestyles (e.g. drinking, smoking) before the confirmed diagnosis were included. The disease conditions among the family members of the 103 participants were also investigated, including the total number of HAE patients and death toll in their family.

### Statistical analysis

All the clinical results were obtained from medical records and the above questionnaire.

The data for exploring the natural course of HAE composed of two parts: one came from the participants who had never adopted any prophylactic drug for HAE; the other was from the patients with a history of medication, but only the periods before they got confirmed diagnosis and received medications were analyzed to avoid the drug intervention. Patients were classified and counted by the disease status they chose at each age stage. We scored “first episode” and “exacerbation stage” three points, “stable stage” two points, “silent stage” and “remission stage” one point. The weighted average of each age stage was calculated to reflect the overall development of HAE.

We proposed a new severity scoring system to evaluate the severity of HAE before confirmed diagnosis according to the edema frequency and locations to explore the possible risk factors. The sum of the average annual episode frequency multiplied by the corresponding coefficient was used as the severity score. Although skin edema has a high incidence and affects people’s mood and daily work, it seldom causes serious clinical symptoms or threatens the lives of patients, leaving the impression that patients need no treatment. Therefore, the frequency of skin edema was multiplied by one. Cases presenting gastrointestinal or laryngeal angioedema but without the need for emergency visits were multiplied by two and with the requirement for emergency treatment by three. Mann-Whitney U test was used to evaluate the possible risk factors according to the severity score and mean edema resolution time, including gender, BMI (body mass index), smoking and alcohol. Statistical analysis was performed by IBM SPSS Statistics 25 software.

## Results

### Demographic and clinical characteristics

One hundred and three patients (including 47 males and 56 females) with a mean age of 37.7 years (range, 15 to 68) were included, shown in Table 1. Seventeen patients in our survey have never adopted danazol or any other prophylactic agent for HAE, among whom 41.18% worried about side effects, 47.06% presented with very mild symptoms and 17.65% found the drug unavailable nearby. Of the remaining 86 patients, the disease development before they received a confirmed diagnosis and no drugs for HAE were also analyzed. The median diagnostic delay was 11 (interquartile range [IQR], 6–19.5) years. Before the diagnosis, the median frequency of skin, laryngeal and gastrointestinal edema was 6 (IQR, 3–14), 0.5 (IQR, 0–2) and 2 (IQR, 0–5) times per year, respectively. The median severity score was 23 (IQR, 9.5–45) and the median edema resolution time was 72 (IQR, 36–72) hours. Of the 103 patients we surveyed, 223 family members were reported to share similar angioedema symptoms and 30 have died of laryngeal edema to date, with the median age of death of 46 (IQR, 35–53) years old, indicating that HAE may severely affect the lifespan of patients.

**Table 1:** Demographics and clinical characteristics of HAE attacks

**Patient demographics (*N*** **= 103)**	
Age, mean (range) (yr)	37.7 (15–68)
Gender, N	
Male	47
Female	56
Have a drinking history, N	45
Male	33
Female	12
Have a smoking history, N	29
Male	25
Female	4
**Edema Characteristics**	
Age of first episode, mean (range) (yr)	17.5 (1–40)
Annual Frequency, median (IQR)	
Skin	6 (3–14)
Gastrointestinal tract	2 (0–5)
Requirement for emergency treatment	1 (0–4)
Laryngeal	0.5 (0–2)
Requirement for emergency treatment	0.25 (0–1)
Severity score, median (IQR)	23 (9.5–45)
Edema resolution time, median (IQR) (h)	72 (36–72)
Diagnostic delay, median (IQR) (yr)	11 (6–19.5)

### Development of HAE

**Fig. 1:**
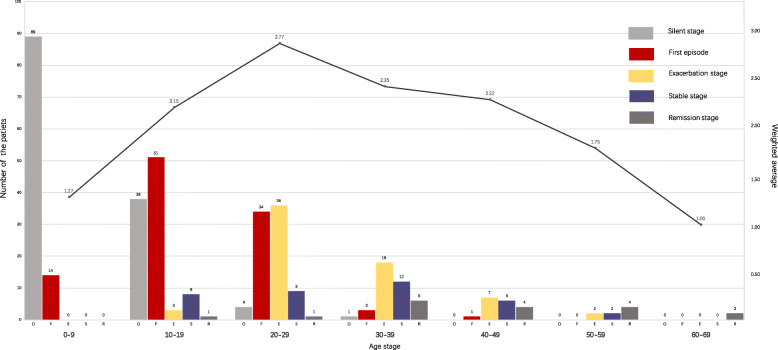
The natural course of HAE over the seventy years. Score first episode *(F)* and exacerbation stage *(E)* 3 points, stable stage *(S)* 2 points, silent stage *(O)* and remission stage *(R)* 1 point. Distribution of the patients with different HAE progression types at each age stage was shown by cluster bar. The line reflected the changes of weighted average over the seventy years

The number of patients with different disease progression patterns at each age stage is shown in Table 2 and Fig. 1.

**Table 2:** Patients with different disease progression patterns at each age stage

Age stage	Patients, N	Patients under therapy-free conditions							Excluded^**b**^
		Included, n	Silent	First episode	Exacerbation	Stable	Remission	Weighted average^**a**^	
0–9	103	103	89	14	0	0	0	1.27	0
10–19	103	101	38	51	3	8	1	2.15	2
20–29	95	84	4	34	36	9	1	2.77	11
30–39	74	40	1	3	18	12	6	2.35	34
40–49	36	18	0	1	7	6	4	2.22	18
50–59	18	8	0	0	2	2	4	1.75	10
60–69	4	2	0	0	0	0	2	1.00	2

^a^Weighted average = (number of the patients at silent or remission stage ×1+ number of the patients at stable stage ×2+ number of the patients with first episode or at exacerbation stage × 3) / n

^b^ Patients were excluded from the analysis because of receiving a confirmed diagnosis and prophylactic drugs at this age stage

#### Age of onset

Among the 103 patients, the mean age of the first episode of HAE was 17.5 (range, 1 to 40) years old. Fourteen (13.6%) patients had their first HAE attack before 10 years old, about half (51/103, 49.5%) of the patients at the age stage of 10 to 19 and one third (34/103, 33.0%) during their twenties. In our survey, the first episode of HAE occurred in three patients over 30 years old and one patient presented no symptoms until 40.

#### Disease progression

The weighted average of the seven age stages was 1.27, 2.15, 2.77, 2.35, 2.22, 1.75, 1.00 respectively, shown in Fig. 1, which may generally reflect the overall natural course of HAE. Among the 95 patients during the age stage of 20 to 29, 11 were excluded from statistics for receiving a diagnosis and taking the prophylactic drug, and of the remaining 84 patients, HAE firstly occurred in 40.5% (34/84) of them and worsened in 42.9% (36/84), indicating that the disease deteriorated for most cases in their twenties. With the growth of age, the proportion of the patients whose conditions were alleviated increased gradually and was 0 (0/103, age 0–9), 1.0% (1/101, age 10–19), 1.2% (1/84, age 20–29), 15% (6/40, age 30–39), 22.2% (4/18, age 40–49), 50% (4/8, age 50–59) and 100% (2/2, age 60–69) respectively in order. After 30 years old, the weighted average decreased along with aging but maintained relatively severe (above 2) before 50.

### Exploration of possible risk factors

The possible risk factors including gender, BMI, alcohol, and smoking were explored according to the severity score and edema resolution time, shown in Table 3. Mann-Whitney U test was run to determine if these factors could affect HAE severity. Distributions of the severity scores or edema resolution time were similar between the groups being compared, as assessed by visual inspection. The medians of severity score and edema resolution time for females were both higher than males but were not statistically significantly different. Only males were considered when exploring the effects of alcohol and smoking due to the rarity of smokers and drinkers among female patients. Seven participants in our survey reported that alcohol could trigger an attack of HAE. However, no statistically significant difference was observed between drinkers and non-drinkers, and interestingly, HAE seems less serious in patients with a history of drinking according to the medians of score and edema resolution time. The correlations between HAE severity and BMI or smoking are also not significant.

**Table 3:** Possible risk factors affecting HAE natural course

**Risk factors**	**Patients (*N*** **= 103)**	**Severity score**		**Mean edema resolution time**	
**Gender**					
Female	56	30.25 (11.25–45)	*P* = 0.067	72 (37–74.25)	*P* = 0.179
Male	47	15 (6.1–40)		60 (36–72)	
**BMI**					
< 25	48	23.25 (10–43)	*P* = 0.937	72 (48–78)	*P* = 0.081
> 25	55	23 (9–50)		48 (36–72)	
**Risk factors**	**Males (*N*** **= 47)**	**Severity score**		**Mean edema resolution time**	
**Alcohol**					
Yes	33	14 (7–25.25)	*P* = 0.139	48 (36–72)	*P* = 0.287
No	14	36.5 (6.075–70.75)		72 (35–78)	
**Smoking**					
Yes	25	15 (5.5–30.75)	*P* = 0.337	48 (36–72)	*P* = 0.467
No	22	17.5 (9–52.125)		66 (36–74)	

## Discussion

Our study collected the data from 103 type 1 HAE patients to analyze the natural course of HAE and explore possible risk factors. Two hundred and thirty-three family members with HAE of these 103 participants were included for the death statistics.

Patients with type 2 HAE were not included in this survey. According to the epidemiological research conducted in our center in 2018 [13] (included the population surveyed in this study) and 2012 [10] (17 patients in this study involved), the percentage of type 2 HAE patients in China is shown less than 5% and far below the expected 15–20%. The number of the type 2 case is relatively small, which is not sufficient to explore the difference between type 1 and type 2 in the natural course. The exclusion of type 2 patients ensured the homogeneity of the study subjects to some extent.

Our results show that under therapy-free conditions, the disease may firstly worsen as the age increased and showed a notable sign of deterioration especially during the age stage of 20 to 29. Then the symptoms were gradually relieved but maintained relatively severe until 50 years old. Patients may die of asphyxia due to the abrupt laryngeal edema with the median age of 46 (IQR, 35–53) years old. Consequently, although the use of danazol was sometimes rejected by patients, especially females, for the common adverse effects, including weight gain, virilization, myalgia, menstrual irregularities, headache, liver dysfunction, hypertension, and psychological abnormalities [14, 15], it is still recommended to conduct long-term prophylaxis of danazol, especially for young and middle-aged patients, because of other medications for prophylaxis are not available now in our country. However, attenuated androgen is thought to be contraindicated among pregnant and lactating women or patients under 16 years old in our clinical practice.

The disease natural progression may provide some new insight to its pathogenesis. C1-INH-HAE results from the uncontrolled release of bradykinin from high molecular weight kininogen (HMWK) due to the deficiency of functional C1-INH. Previous studies have observed the level of kininogen increased with age in rat serum [16–19]. However, aging leads to weaker responsiveness to kinins [8] which is believed to be realized by the decreased density of the bradykinin receptors, lessening the vasoactive effects of bradykinin on the heart and vasculature [20–22]. And interestingly, vasodilation induced by bradykinin can be transformed into vasoconstriction under the conditions of vascular disorder or inflammation which is increased by aging and hypertension, a common chronic disease in older patients [23]. These findings may explain why the edema attacks caused by bradykinin turned to alleviate along with aging.

Our survey shows that nearly half of the patients had their first HAE attack during puberty and one third during their twenties. High levels of sex hormones may be one of the reasons why HAE deteriorates in youth. It is known that HAE is influenced by the fluctuation of estrogen [24, 25]. Combined (estrogen and progestogen) oral contraceptives also has been found to worsen HAE for 80% of female patients [25]. Previous studies at molecular levels have confirmed that estrogen can increase the generation of kallikrein and bradykinin [26, 27] and plays a role in the regulation of B2 receptor gene expression [28]. In a 2006 survey [2], more than 12 attacks of HAE occurred per year in 71 of 117 women (60.7%) and 34 of 78 men (43.6%), and the difference was significant (*P* < 0.020). In our study, both the median severity score and median edema resolution time of female patients were higher than those of male patients, but the difference was still not significant enough, with a *P* value of 0.067 and 0.179, respectively. Maybe the sample size needs to be expanded further.

Seven drinkers in our survey reported that alcohol could trigger an attack of HAE, and similar result was also reported before [29]. Alcohol is known to potentiate the action of bradykinin [30]. However, no significant difference was observed between the drinkers and non-drinkers. And interestingly, the median severity score and edema resolution time were lower in drinkers, which seem contrary to the previous description. Endothelial function and low-grade vascular inflammation are associated with dietary intake including alcohol [31]. Red wine consumption has been reported to cause less endothelial dysfunction and reduce inflammation in vasculature [32] while other studies found that heavy or even light alcohol consumption were associated with impaired endothelial function [33, 34]. The relationship between the alcohol and endothelia is complicated, which may involve the type and amount of alcohol consumed, the age and race of the drinker, and many other factors. And how the endothelial dysfunction or inflammation affects the vascular permeability is also worth much more research.

We did not find other relative risk factors that may affect HAE natural progression, including smoking, though active and passive smoking has been found to blunt endothelium-dependent vasodilatations [35]. It may be due in part to the imperfect methods of scoring HAE severity. The disease severity is difficult to classify mainly for the changeability of symptoms, interference of subjective perception and no validated biomarkers [36]. And every HAE patient is at a risk for unpredicted laryngeal edema and death by asphyxiation, regardless of past symptoms, making the severity assessment more difficult. Based on the above reasons, we scored the severity according to edema frequency and locations to reduce the interference caused by changes over time or personal perceptions. But the scoring system is still not sufficient in its current form, and more researches are needed for the development of validated tools.

## Conclusions

In conclusion, this study was dedicated to the natural course of HAE throughout patient life under therapy-free conditions. HAE showed a notable sign of exacerbation especially during the age stage of 20 to 29 and was alleviated with the growth of the age, presenting fewer episodes or milder symptoms. Patients of all ages are recommended to conduct active long-term prophylaxis and have drugs prepared in case an acute episode occurs, because some patients may die of asphyxia due to acute laryngeal edema. The prophylactic and therapeutic strategy might be adjusted depending on the disease progression as aging. No direct evidence has been obtained to prove HAE severity is significantly related to gender, BMI, alcohol or smoking. We hope this study may provide some clues for the clinical practice.

## Data Availability

The datasets generated and analyzed during the current study are not publicly available but are available from the corresponding author on reasonable request.
